# Race, Ethnicity, and Linguistic Isolation as Determinants of Participation in Public Health Surveillance Surveys

**Published:** 2005-12-15

**Authors:** Michael W Link, Ali H Mokdad, Herbert F Stackhouse, Nicole T Flowers

**Affiliations:** Centers for Disease Control and Prevention; Centers for Disease Control and Prevention, Atlanta, Ga; Centers for Disease Control and Prevention, Atlanta, Ga; Centers for Disease Control and Prevention, Atlanta, Ga

## Abstract

**Introduction:**

To plan, implement, and evaluate programs designed to improve health conditions among racial and ethnic minority populations in the United States, public health officials and researchers require valid and reliable health surveillance data. Monitoring chronic disease and behavioral risk factors among such populations, however, is challenging. This study assesses the effects of race, ethnicity, and linguistic isolation on rates of participation in the Behavioral Risk Factor Surveillance System (BRFSS).

**Methods:**

County-level data from the 2003 BRFSS survey and 2000 U.S. census were used to examine the effects of race, ethnicity, and linguistic isolation on six measures of survey participation (i.e., rates of resolution, screening, cooperation, response, language barriers, and refusal).

**Results:**

Participation rates were significantly lower in counties with higher percentages of black people and people who did not speak English. Response rates decreased by 4.6% in counties with the highest concentration of black residents compared with counties with few black residents. Likewise, response rates decreased by approximately 7% in counties in which a larger percentage of the population spoke only Spanish or another Indo-European language compared with counties in which all residents spoke English.

**Conclusion:**

The negative relationship between the percentage of Spanish-only–speaking households and participation rates is troubling given that the BRFSS is conducted in both Spanish and English. The findings also indicate that more needs to be done to improve participation among other minorities. Researchers are investigating several ways of addressing disparities in participation rates, such as using postsurvey adjustments, developing more culturally appropriate data-collection procedures, and offering surveys in multiple languages.

## Introduction

Reducing racial and ethnic disparities in health is an overarching goal of *Healthy People 2010* (1). To reach this goal, however, public health officials require valid and reliable data from health surveillance to plan, implement, and evaluate programs designed to improve health conditions among racial and ethnic minority populations (2-4). For instance, health surveillance efforts have highlighted racial and ethnic disparities in health conditions such as cardiovascular disease, hypertension, diabetes, certain cancers (e.g., colon and rectal, pancreatic, stomach), and nationally notifiable diseases (e.g., chlamydia, gonorrhea, salmonelosis) and in risk factors for chronic conditions such as physical inactivity, excessive alcohol consumption, and cigarette smoking (5-11). Despite the success of many surveillance efforts, monitoring chronic disease and behavioral risk factors among minority populations remains a challenge.

The proportion of racial and ethnic minorities who participate in major health surveys is often lower than the proportion for the overall U.S. population. Some of the reasons for lower rates of participation among racial and ethnic minorities include disproportionate mistrust of government and the research community, cultural and language barriers, lower rates of literacy and health literacy (the capacity to obtain, process, and understand basic health information and services needed to make appropriate health decisions), high mobility patterns, reluctance to reveal personal information, and data-collection procedures (e.g., characteristics of the interviewers) (12-26). Because minority groups, particularly groups of lower socioeconomic status, may be underrepresented in public health statistics generated by these surveys, the health risks and health problems that they face may be inadequately described.

**Figure 1:**
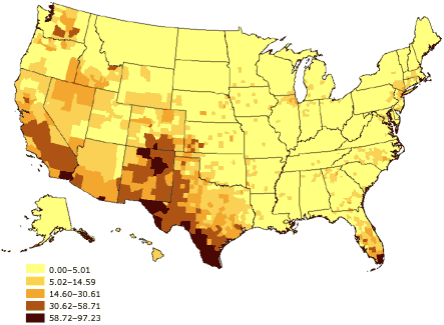
Percentage of Hispanic or Latino adults aged 18 years and older, United States. Source: U.S. Census 2000 (32).

**Figure 2:**
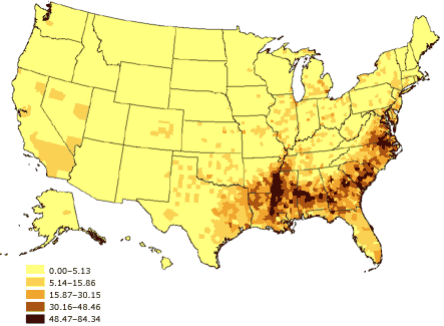
Percentage of black or African American adults aged 18 years and older, United States. Source: U.S. Census 2000 (32).

**Figure 3:**
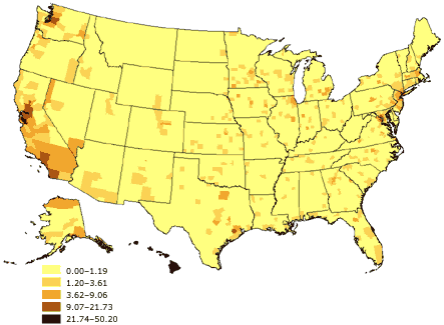
Percentage of Asian or Pacific Islander adults aged 18 years and older, United States. Source: U.S. Census 2000 (32).

The potential for such problems has increased over the past several decades as the U.S. population has grown more diverse. From 1980 to 2002, according to the U.S. Census Bureau, the proportion of minorities among the civilian, noninstitutionalized population grew from 6.4% to 13.3% among Hispanics, 11.7% to 13.0% among blacks, and 1.5% to 4.4% among Asians (27-30). Additionally, as of 2002, 11.7% of U.S. residents were reported to have been born in a foreign country, with 53.3% of those saying they had been born in Latin America, 25.0% in Asia, 13.7% in Europe, and 8.0% in some other region of the world (31). Moreover, the various racial and ethnic groups are not distributed equally across the United States (Figures 1–3). As a result, the potential impact of race and ethnicity on survey participation rates varies considerably among and within regions.

**Figure 4:**
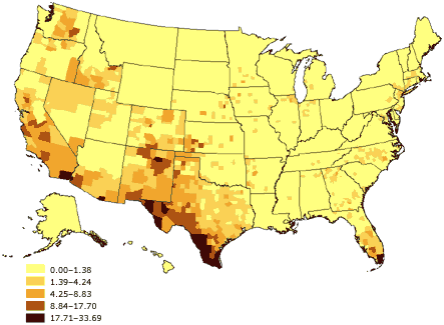
Percentage of linguistically isolated Spanish-language households, United States. Source: U.S. Census 2000 (32)

**Figure 5:**
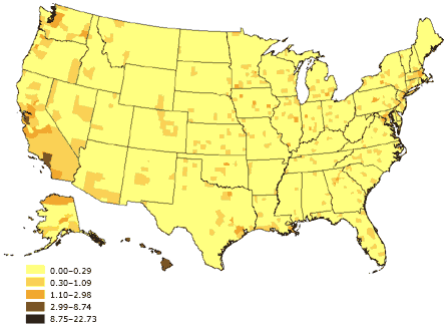
Percentage of linguistically isolated Asian-language or Pacific Island-language households, United States. Source: U.S. Census 2000 (32).

**Figure 6:**
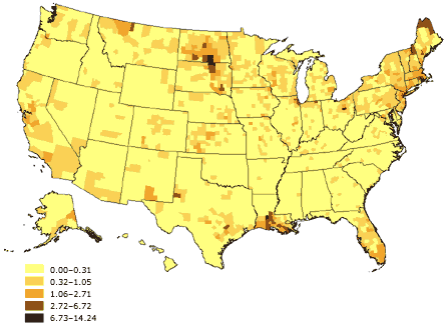
Percentage of linguistically isolated Indo-European–language households, United States. Source: U.S. Census 2000 (32).

There has also been a corresponding growth in the percentage of U.S. residents who primarily speak a language other than English. According to the 2000 census, 47.0 million (18%) of the 262.4 million people aged 5 years and older spoke a language other than English at home (33). This percentage increased from 14% in 1990 and from 11% in 1980. *Linguistic isolation* is defined by the U.S. Census Bureau as living in a household in which all members aged 14 years and older speak a non-English language and also speak English less than "very well" (i.e., have difficulty with English) (32). In 2000, approximately 4.5% of the U.S. population could have been considered linguistically isolated. Among certain subpopulations, however, the percentage of people who said they spoke English less than very well was high: 51% of individuals spoke an Asian or Pacific Island language, 49% spoke Spanish, and 34% spoke another Indo-European language (33). Table 1 shows the major languages included in each group. Again, the types of languages spoken within linguistically isolated households across the United States vary by region (Figures 4–6). Because most health surveys are typically conducted in English only, linguistic isolation can be expected to significantly increase the level of nonresponse among people who do not speak English.

**Figure 5.** Percentage of linguistically isolated Asian-language or Pacific Island-language households, United States. Source: U.S. Census 2000 (32).

As one of the world's largest health surveillance systems, the Behavioral Risk Factor Surveillance System (BRFSS) has been instrumental in tracking health disparities across populations in the United States (34). Yet over the past decade, BRFSS participation rates, like those of most other surveys, have declined sharply (35). As part of an effort to reverse this trend and ensure the reliability and validity of BRFSS data, we assessed the impact of race, ethnicity, and linguistic isolation on measures of survey participation. This report includes the results of that assessment as well as a discussion of potential means of improving survey participation rates among these groups, thus making population-based health surveys like the BRFSS surveys more representative of the entire population.

## Methods

The BRFSS gathers data through computer-assisted telephone interview (CATI) surveys designed to collect uniform, state-specific data on preventive health practices and risk behaviors that are linked to the leading causes of morbidity and mortality among adults. The survey is conducted by all 50 states and the District of Columbia, as well as by Puerto Rico, Guam, and the Virgin Islands with assistance from the Centers for Disease Control and Prevention (CDC). However, the three territories are not included in the analysis presented here. Further details on the BRFSS design, methodology, and questionnaire are presented elsewhere (36).

### Measures and variables

To examine aspects of survey participation, we calculated the following six dependent measures of survey participation at the county level based on final case disposition for telephone numbers called between January 1, 2003, and December 31, 2003:

1. Resolution rate: the percentage of all sampled telephone numbers for which household status with a working telephone number has been determined

2. Screening rate: the percentage of all known households in which the presence or absence of an eligible respondent has been determined

3. Cooperation rate: the percentage of known, eligible households in which a completed or partially completed interview has been obtained

4. Response rate: the percentage of all confirmed and potentially eligible sample members for whom an interview has been completed, which we calculated using Response Rate 4 recommended by the American Association for Public Opinion Research (37)

5. Language-barrier rate: the percentage of all sampled households given a final disposition of *language barrier.* Interviewers could not communicate with household members because of the language spoken in the home (which was presumably not English or Spanish, the two languages in which the BRFSS survey is conducted)

6. Refusal rate: the percentage of all sampled households given a final disposition of *refusal*, indicating either the selected sample member's refusal to complete the interview or the interviewer's inability to recontact the household because of a hang-up or other refusal by someone in the household

We conducted the analysis at the county level because of a lack of available information about survey nonrespondents at the individual or household level. Counties were included in the analysis if they had 30 or more observations in the denominator of each of the six participation measures. Our use of these criteria ensured greater stability in the measures calculated and helped us compare the impact of independent variables across the six models estimated; however, it also limited the analysis to 1894 of the 3141 counties in the United States.

County-level predictor variables for race, ethnicity, and linguistic isolation were derived from 2000 U.S. census counts (32). We calculated the percentage of each county's population that was black or African American, Asian, and Hispanic, as well as the percentage who spoke only Spanish, only an Asian language, or only another Indo-European language. On average, the included counties had somewhat higher percentages of blacks than did the nonincluded counties (9.0% compared with 7.1%), Asians (1.2% compared with 0.4%), Asian-language–only households (0.2% compared with 0.1%), and Indo-European–language–only households (0.4% compared with 0.3%) but slightly lower percentages of Hispanics (4.8% compared with 6.9%) and Spanish-language–only households (1.0% compared with 1.5%).

We also developed several county-level control variables to account for some of the other factors that are thought to affect participation rates within certain geographic areas. Socioeconomic status, often measured through a combination of income and education levels, is an important mediator of racial and ethnic health disparities and an important predictor of survey participation (2,38). Likewise, living in an urban area, being away from home frequently, and screening calls with answering machines, caller-identification devices, or similar devices have been shown to reduce respondent contactability and participation rates (21,38,39)We also developed several county-level control variables to account for some of the other factors that are thought to affect participation rates within certain geographic areas. Socioeconomic status, often measured through a combination of income and education levels, is an important mediator of racial and ethnic health disparities and an important predictor of survey participation (2,38). Likewise, living in an urban area, being away from home frequently, and screening calls with answering machines, caller-identification devices, or similar devices have been shown to reduce respondent contactability and participation rates (21,38,39). We used 2000 U.S. census data to develop four control variables based on 1) the percentage of households in each county with incomes of $50,000 or more, 2) the percentage of adults aged 25 and older in each county who had less than a high school education (e.g., no high school diploma or equivalency), 3) the percentage of households in each county that were in urban areas, and 4) the percentage of households in each county with heads of household who had a one-way commute to work of 30 minutes or more. We used BRFSS data to calculate a fifth control variable measuring the percentage of all calls made within a county that resulted in contact with an answering machine, privacy manager, or some other identifiable type of call-screening device. 

### Statistical analysis

Because all variables in the analysis were expressed as percentages, we used ordinary least squares (OLS) regression modeling to assess the impact of race, ethnicity, and linguistic isolation on survey participation. In preliminary analyses, we found a high degree of correlation between Asian race and Asian-language isolation (r = 0.92; *P* < .001) and between Hispanic ethnicity and Spanish-language isolation (r = 0.93; *P* < .001). We used separate models to determine which variables (race, ethnicity, or linguistic isolation) were better predictors, but we found the differences between them to be marginal. Although linguistic isolation is a definite barrier to survey participation, race and ethnicity may or may not be factors; thus, the Asian-language–only and Spanish-language–only variables were retained in the final models, but the variables Asian race and Hispanic ethnicity were not retained. Because of the strong correlation between race, ethnicity, and language-isolation variables, however, we had difficulty determining the proportional impact of each.

We also examined the possible effects of multicollinearity in our analysis. Because we found that regressing the other predictor and control variables on urbanicity explained more than 50% of the variance in the percentage-urban variable (adjusted R^2^ = 0.52; F = 296.7), we removed urbanicity from the final models.

The final OLS models were estimated for each of the six participation measures (rates of resolution, screening, cooperation, response, language barriers, and refusal). The dependent variables used were the county-level estimates for percentage of black adults, percentage of Spanish-language–only households, percentage of other Indo-European-language–only households, and percentage of Asian-language–only households. The control variables used were the percentage of households with incomes of $50,000 or more, the percentage of adults aged 25 and older with less than a high school education, the percentage of heads of household with a one-way work commute of 30 minutes or more per day, and the percentage of BRFSS calls that reached an answering machine or call-screening device. Model selection was based on forced entry of all variables into the models rather than stepwise selection. The models were estimated using SPSS 13.0 (SPSS Inc, Chicago, Ill) with the Complex Samples module. 

Finally, we used the OLS coefficients from the final models and the maximum county-level population parameters to calculate the maximum impact of race, ethnicity, and linguistic isolation on the six measures of survey participation.

## Results

In general, minority race and ethnicity and linguistic isolation had significant negative correlations with survey participation rates (Table 2). The regression coefficients (β) in these models estimate the amount of increase or decrease in the dependent measures for every one-unit difference in the independent variables. For example, for every percentage-point increase in the black population of a county, the county-level response rate declined by 0.06%. Counties with higher percentages of black residents tended to have significantly lower rates of participation and higher refusal rates. Statistically significant (α = .05) negative relationships were noted between the percentage of black adults in a county and county-level resolution rates (β = −0.03; *P* = .001), screening rates (β = −0.18; *P* = .001), cooperation rates (β = −0.13; *P* = .001), and response rates (β = −0.06; *P* = .001), although a significant positive relationship was seen with refusal rates (β = 0.06; *P* = .001). The percentage of black residents in a county did not have a significant effect on the rate of nonparticipation attributed to a language barrier.

Linguistic isolation also had a negative effect on participation rates, although the magnitude of this effect differed across the three language types. Higher rates of Spanish-language isolation led to lower resolution rates (β = −0.11; *P* = .04), screening rates (β = −0.92; *P* = .001), cooperation rates (β = −0.58; *P* = .001), and response rates (β = −0.26; *P* = .001). The impact of Spanish-language isolation on response rates was more than four times the impact of the percentage of black adults in a county. Counties with higher percentages of Spanish-language–only households also had higher percentages of nonparticipation attributed to language barriers (β = 0.12; *P* = .001). Rates of Spanish-language isolation did not, however, significantly affect the percentage of nonparticipation attributed to refusals.

The percentage of households in which only Indo-European languages were spoken did not significantly affect resolution rates, but it did have a significant negative effect on screening rates (β = −1.36; *P* = .001), cooperation rates (β = −1.39; *P* = .001), and response rates (β = −0.64; *P* = .001). Counties with higher rates of Indo-European-language–only households also had higher language-barrier and refusal rates.

In contrast, Asian-language–isolated households had less effect on survey participation rates. Counties with higher percentages of Asian-language–only households did have significantly lower resolution rates (β = −0.48; *P* = .03) and screening rates (β = −0.98; *P* = .03), but they did not have significantly lower cooperation or response rates. Similarly, although higher percentages of Asian-language isolation led to a significant increase in the language-barrier rate (β = 0.14; *P* = .001), there was a significant decrease in the refusal rate in these counties (β = −1.90; *P* = .001).

Overall, race, ethnicity, and language-isolation models explained 27% to 31% of the variance in the screening, response, and language-barrier rates, but the models were only about half as effective in explaining variance in resolution, cooperation, and refusal rates.

Because the impact of race, ethnicity, and linguistic-isolation variables depended on the size of a county subpopulation, we calculated the maximum impact of these variables among the subset of 1894 counties examined here. Table 3 shows the amount of change we might expect in the percentage of each rate in counties with the highest concentrations of black residents and language-isolated households. We calculated this expected change by multiplying the high range value for each population characteristic by its corresponding OLS coefficient from Table 2. We found, for example, that in counties in which slightly more than one fourth of the households spoke only Spanish, screening rates were approximately 25% lower than in counties with no Spanish-language–only households. We also found that in counties in which approximately three fourths of the adult population was black, response rates were 5% lower than in counties with no black residents. Response rates were 7% lower in counties with the highest concentrations of households in which Spanish was the predominant language and no one in the household spoke English very well. Likewise, response rates were approximately 7% lower in counties with higher concentrations of households in which other Indo-European languages were spoken rather than English.

## Discussion

Our study revealed that survey participation rates were significantly lower in areas with higher concentrations of racial and ethnic minorities and linguistically isolated households. These important findings indicate the need to ensure adequate representation of these populations in large-scale health surveys such as the BRFSS. As we examine ways of increasing BRFSS participation rates, these findings will help us to design and implement more effective means of involving these hard-to-reach populations.

One particularly disturbing finding was the significant impact of Spanish-language isolation on participation rates, given that BRFSS surveys are offered in both Spanish and English. Education is an important mediating factor in survey participation among Hispanic individuals because lower levels of literacy and health literacy have been related to a greater reluctance by Hispanic individuals to participate in health surveys (26,40). Our study shows, however, that even after controls are added for education, areas with higher concentrations of Spanish-only–speaking households are less likely to participate in health surveys. This may be because of ineffective procedures for contacting and eliciting participation from predominantly Spanish-speaking households, lack of bilingual or Spanish-speaking interviewers, or inadequate training of Spanish-speaking interviewers. It is also likely that current Spanish-language survey translations do not adequately address the different Spanish dialects spoken in the United States, such as those spoken by individuals or families originating from Mexico, Puerto Rico, or Cuba (41). Moreover, it may also reflect the impact of ethnic and cultural issues. Therefore, we may have to assume that concepts and interpretation are culturally dependent (42,43). We were unable, however, to disentangle the influence of language and culture.

Our findings also indicate that more needs to be done to improve participation among other minorities, such as African Americans, Asians who are isolated by language, and other linguistically isolated groups. To this end, researchers are investigating ways to address disparities in participation rates by postsurvey adjustments, culturally appropriate data-collection procedures, and multiple language use.

Standard techniques are widely used to compensate for demographic differences between a survey sample and the general population it represents (21). Postsurvey adjustments such as weighting and stratification represent standard practices in most major health surveys. However, these techniques are often limited to a few key demographic variables for which population estimates are available. Moreover, they may produce larger standard errors that decrease the precision of estimates.

Researchers need to develop survey designs that better address the increasingly complex racial, ethnic, and linguistic mix of the U.S. population. A U.S. Department of Health and Human Services report recommended that "culturally and linguistically appropriate interviewing techniques need to be employed at all times when conducting surveys on racial and ethnic issues" (4). The report further recommended that relevant cultural factors and language requirements be incorporated into survey designs when feasible. Researchers need to be cognizant of the customs, values, and beliefs of individuals in minority communities, particularly because they relate to the sharing of personal information, including health care practices and health conditions (44). Focus groups and cognitive interviews of people from various backgrounds can help determine whether respondents will interpret and respond to survey requests and questions as intended (45,46).

Some research has shown that the race, ethnicity, and sex of an interviewer can affect a respondent's level of cooperation (14). Because an interviewer with a background and characteristics similar to those of a potential survey participant may not be available, it is important that interviewers be trained to understand and manage multiple culturally specific issues. This understanding requires the development and implementation of cultural-sensitivity training programs for interviewers. Culturally specific scripts could also be made available to interviewers in anticipation of challenging situations.

Researchers also need to consider increasing the number of languages in which a survey is offered, especially in communities where rates of linguistic isolation are high. Moreover, it is important to ensure that the translated questions are culturally equivalent in terms of coherence and appropriateness (19,20,47,48).

Researchers have used two approaches in addressing linguistic isolation. The first approach is to translate the questionnaire and hire interviewers who are fluent in that language. This native-language speaker approach is used by the California Health Interview Survey (CHIS), a state-based telephone survey similar in content to the BRFSS survey. The 2001 CHIS was translated into Spanish, Mandarin Chinese, Cantonese, Vietnamese, Korean, and Cambodian (Khmer). Approximately 10% of the completed interviews in the state were conducted in Spanish, and 5% were conducted in one of the Asian languages (49).

The second approach is to rely upon third-party interpreters to administer the survey. Some language-service providers can provide interpreters in more than 150 languages (50). Using a three-way telephone connection, for example, an interpreter (who has access to an English version of the questionnaire but not necessarily a version translated into the respondent's language) translates the conversation between the English-speaking interviewer and the native-language–speaking respondent. This approach is used for the National Immunization Survey (NIS), a telephone survey that collects immunization information on children aged 19 to 35 months living in U.S. households. In 2002, interviews conducted by this method accounted for 4% to 5% of the completed interviews in areas such as Boston, Newark, New York City, and King County, Wash (51).

Both of these translation approaches have advantages and disadvantages. The use of native-language–speaking interviewers helps ensure that the survey questionnaire is administered in a standardized manner but reduces the number of languages and interviewers available. In contrast, third-party interpretation allows questionnaires to be administered in many languages, but administration of the questionnaire may be less consistent. Third-party interpretation also does not allow for assessment of cultural equivalence, thereby potentially leading to measurement error. Both approaches are also relatively costly. Additionally, neither approach provides a complete solution to the problem of increasing survey participation among people isolated by language.

There are several limitations to the current study. First, sample sizes in some counties limited the analysis to 1894 of 3141 counties. Second, because information on key variables such as race, ethnicity, and language spoken in the household was not available at the individual level of nonresponding households, the analysis was conducted at an aggregate (county) level. Although aggregate-level approaches to studying racial and ethnic disparities have been encouraged by the U.S. Department of Health and Human Services when individual-level data are not available, future studies of survey participation could be strengthened by surveys that are designed to collect data on key variables from nonrespondents (4). Third, the high correlation between the race, ethnicity, and language variables for Asian and Hispanic individuals limited our ability to disentangle the effects on survey participation of culture and language for these two groups.

Adequately identifying racial and ethnic disparities in health care and developing effective strategies to eliminate these disparities depends on the availability of valid and reliable data. Considerations of race, ethnicity, acculturation, and language are critical to the success of such health surveillance efforts. Researchers need to infuse these elements into their study designs, data-collection protocols, and data-processing routines. Indeed, several pilot studies are now being conducted in conjunction with the BRFSS to try to address these issues. These studies are using alternative sampling frames to reach individuals who are inaccessible by landline telephones, multiple modes of survey data collection, prenotification techniques tailored to minority racial and ethnic populations, surveys in languages other than English and Spanish, and case-management techniques for preassigning likely non-English–speaking households to bilingual interviewers. Such efforts are essential for meeting the challenges to health surveillance posed by the growing diversity of the U.S. population.

## Figures and Tables

**Table 1:** Major Languages Spoken by English-Language–Isolated Groups^a^

**Language Group**	**Major Languages**
Spanish	Spanish, Ladino
Other Indo-European languages	Most languages of Europe and the Indic languages of India, including the Germanic languages, such as German, Yiddish, and Dutch; the Scandinavian languages, such as Swedish and Norwegian; the Romance languages, such as French, Italian, and Portuguese; the Slavic languages, such as Russian, Polish, and Serbo-Croatian; the Indic languages, such as Hindi, Gujarathi, Punjabi, and Urdu; Celtic languages; Greek; Baltic languages; and Iranian languages.
Asian and Pacific Island languages	Chinese; Korean; Japanese; Vietnamese; Hmong Khmer; Lao; Thai; Tagalog or Pilipino; the Dravidian languages of India, such as Telegu, Tamil, and Malayalam; and other languages of Asia and the Pacific, including the Philippine, Polynesian, and Micronesian languages.

a Source: Shin and Bruno (33).

**Table 2:** Effects of Race, Linguistic Isolation, and Other Variables on County-level Participation Rates (N = 1894), 2003 Behavioral Risk Factor Surveillance System

**Characteristic**	**Resolution Rate^a^**		**Screening Rate^b^**		**Cooperation Rate^c^**		**Response Rate^d^**		**Language-Barrier Rate^e^**		**Refusal Rate^f^**	
	**β^g^ (SE)**	***P***	**β^g^ (SE)**	***P***	**β^g^ (SE)**	***P***	**β^g^ (SE)**	***P***	**β^g^ (SE)**	***P***	**β^g^ (SE)**	***P***
% Black residents ≥18 y	−0.03 (0.01)	.001	−0.18 (0.02)	.001	−0.13 (0.01)	.001	−0.06 (0.01)	.001	<0.01 (<0.01)	.07	0.06 (0.01)	.001
% Spanish-language–only households	−0.11 (0.05)	.04	−0.92 (0.11)	.001	−0.58 (0.09)	.001	−0.26 (0.04)	.001	0.12 (0.01)	.001	−0.03 (0.08)	.71
% Asian-language–only households	−0.48 (0.22)	.03	−0.98 (0.45)	.03	−0.24 (0.38)	.53	−0.21 (0.18)	.24	0.14 (0.03)	.001	−1.90 (0.31)	.001
% Indo-European-language–only households	−0.12 (0.15)	.44	−1.36 (0.31)	.001	−1.39 (0.26)	.001	−0.64 (0.12)	.001	0.12 (0.02)	.001	0.82 (0.21)	.001
% Calls that reached answering machines	0.11 (0.01)	.001	−0.19 (0.03)	.001	0.10 (0.02)	.001	0.04 (0.01)	.001	<−0.01 (<0.01)	.001	−0.02 (0.02)	.29
% Households with heads who commute ≥30 minutes one-way to work	−0.04 (0.01)	.001	−0.13 (0.02)	.001	−0.05 (0.02)	.01	−0.05 (0.01)	.001	<0.01 (<0.01)	.02	0.09 (0.02)	.001
% Households with income ≥$50,000	−0.18 (0.01)	.001	−0.30 (0.03)	.001	−0.18 (0.02)	.001	−0.17 (0.01)	.001	<0.01 (<0.01)	.11	0.22 (0.02)	.001
% Adults aged ≥25 y with <high school education	−0.06 (0.02)	.007	−0.06 (0.04)	.16	−0.04 (0.04)	.24	−0.04 (0.01)	.013	<−0.01 (<0.01)	.51	0.15 (0.03)	.001

a Resolution rate = (number of cases determined to be households/total number of cases) ×100%. Intercept: β (SE) = 91.01 (0.81); *P* = .001.  Adjusted R^2^ = 0.18.

b Screening rate = (number of households where eligibility is determined/total number of households) ×100%. Intercept: β (SE) = 93.65 (1.66); *P* = .001.  Adjusted R^2^ = 0.30.

c Cooperation rate = (number of completed interviews/number of confirmed eligible households) ×100%. Intercept: β (SE) = 83.24 (1.38); *P* = .001. Adjusted R^2^ = 0.16.

d Response rate = (number of completed interviews/estimated total number of eligible households) ×100%. Intercept: β (SE) = 43.15 (0.64); *P* = .001. Adjusted R^2^ = 0.31.

e Language-barrier rate = (number of cases with language-problem code/total number of cases) ×100%. Intercept: β (SE) = 0.06 (0.09); *P* =  .54. Adjusted R^2^ = 0.27.

f Refusal rate = (number of cases with refusal code/total number of cases) ×100%. Intercept: β (SE) = 7.31 (1.14); *P* = .001. Adjusted R^2^ = 0.17.

g β indicates ordinary least squares regression coefficient.

**Table 3:** Maximum Impact of Race and Linguistic Isolation on County-level Participation Rates, 2003 Behavioral Risk Factor Surveillance System^a^

**Characteristic**	**Maximum Population Parameter Value^b^(%)**	**β^c^ for Population Characteristic**	**Measures of Survey Participation**					

			**Resolution Rate^d^(%)**	**Screening Rate^e^(%)**	**Cooperation Rate^f^(%)**	**Response Rate^g^(%)**	**Language-Barrier Rate^h^(%)**	**Refusal Rate^i^(%)**
% Black residents −18 y	77.4	−0.03	−2.3	−13.9	−10.1	−4.6	0.0	4.6
% Spanish-language–only households	26.8	−0.11	−2.9	−24.7	−15.5	−7.0	3.2	0.0
% Asian-language–only households	8.7	−0.48	−4.2	−8.5	0.0	0.0	1.2	−16.5
% Indo-European-language–only households	10.8	−0.12	0.0	−14.7	−15.0	−6.9	1.3	8.9

a Impact of variable = maximum county-level population parameter ×β for population characteristic (from Table 1). The impact of variables that were not statistically significant (*P* > .05) in Table 2 are assumed to have no impact and are set to zero in this table.

b Maximum population parameter value = maximum county-level value for population characteristic.

c β indicates ordinary least squares regression coefficient.

d dResolution rate = (number of cases determined to be households/total number of cases) × 100%.

e Screening rate = (number of households where eligibility is determined/total number of households) ×100%.

f Cooperation rate = (number of completed interviews/number of confirmed eligible households) ×100%.

g Response rate = (number of completed interviews/estimated total number of eligible households) ×100%.

h Language-barrier rate = (number of cases with language-problem code/total number of cases) ×100%.

i Refusal rate = (number of cases with refusal code/total number of cases) ×100%.
